# Efficacy of a group tobacco cessation behavioral intervention among tobacco users with concomitant mental illness in Kenya: protocol for a controlled clinical trial

**DOI:** 10.1186/s12889-019-8040-2

**Published:** 2019-12-18

**Authors:** Yvonne Olando, Mary Kuria, Muthoni Mathai, Mark D. Huffman

**Affiliations:** 1 University of Nairobi, Nairobi, Kenya; 2 Northwestern University Feinberg School of Medicine, Chicago, USA; 3 The George Institute for Global Health, UNSW, Sydney, Australia

**Keywords:** Tobacco cessation, Mental illness, Tobacco dependence, Kenya

## Abstract

**Background:**

The rate of tobacco use among people with mental illness is nearly twice that of the general population. Psychotropic medications for tobacco cessation are relatively expensive for most Kenyans. Behavioral counseling and group therapy are effective lower cost strategies to promote tobacco cessation, yet have not been studied in Kenya among individuals with concomitant mental illness.

**Methods/design:**

One hundred tobacco users with mental illness who were part of an outpatient mental health program in Nairobi, Kenya were recruited and allocated into intervention and control groups of the study (50 users in intervention group and 50 users in control group). Participants allocated to the intervention group were invited to participate in 1 of 5 tobacco cessation groups. The intervention group received the 5As (Ask, Advise, Assess, Assist and Arrange) and tobacco cessation group behavioral intervention, which included strategies to manage cravings and withdrawal, stress and anxiety, and coping with depression due to withdrawal; assertiveness training and anger management; reasons to quit, benefits of quitting and different ways of quitting. Individuals allocated to the control group received usual care. The primary outcome was tobacco cessation at 24 weeks, measured through cotinine strips. Secondary outcomes included number of quit attempts and health-related quality of life.

**Discussion:**

This study will provide evidence to evaluate the efficacy and safety of a tobacco cessation group behavioral intervention among individuals with mental illness in Kenya, and to inform national and regional practice and policy.

**Trial registration:**

Trial registration number: NCT04013724.

Name of registry: ClinicalTrials.gov.

URL of registry: https://register.clinicaltrials.gov

Date of registration: 9 July 2019 (retrospectively registered).

Date of enrolment of the first participant to the trial: 5th September 2017.

Protocol version: 2.0.

## Background

Tobacco dependence is recognized as a mental and behavioral disorder in the International Classification of Diseases (ICD-10) of the World Health Organization (WHO) [1] and in the Diagnostic and Statistical Manual of Mental Disorders of the American Association of Psychiatry (DSM-V-TR) [2]. Patients with serious mental disorders have a greater prevalence of tobacco use [3], more severe psychiatric symptoms, poorer overall general well-being, and greater functional impairment when compared to non-tobacco users [4].

‘The likelihood that a person who smokes has a mental disorder is twice that of a person who does not have one’. [5, 6] The proportional relationship between the intensity of the psychiatric symptoms and the severity of the tobacco dependence has already been established [6, 7]. Tobacco-related illnesses including cancer, heart disease, and lung disease are among the most common causes of death among persons with mental illness. Globally individuals with serious mental illness and substance abuse on treatment in the public health system die, on average, 25 years earlier than those without mental illness [8].

According to World Health Organization (WHO), Quality of Life (QOL) definition is individualistic, has a cultural and value systems dimension in relation to the persons goals, expectations, standards and concerns. WHO recognizes 5 domains that should be captured while assessing quality of life: Physical, Psychological, social, environmental, and spiritual [9]. With the rate at which a person with mental illness smokes, they are more likely to suffer from the physical domain, due to the health impact of tobacco use. QOL has been said to involve two dimensions – adaptive functioning (self-care and social roles) and life satisfaction or subjective wellbeing [10]. Most people with mental illness need support to balance their emotional and physical needs.

Randomized controlled trials have shown that tobacco cessation treatment among patients receiving concomitant mental health treatment is effective and safe, does not exacerbate mental health symptoms, and does not lead to increased use of alcohol or illicit drugs [11–13]. However, most research on the efficacy and safety of tobacco cessation interventions has excluded participants with a past or present history of mental illness; thus, this population has remained underrepresented in randomized trials of tobacco cessation intervention strategies [14].

Morris et al. [15] found that most mental health providers receive little or no training on smoking cessation and frequently have misconceptions, such as that patients with mental illnesses need to use tobacco to control their psychiatric symptoms and that these individuals have no desire to quit. A 2013 Cochrane systematic review [16] demonstrated how myths have led to persistently high tobacco use among the mentally ill, including myths that persons suffering psychological disorders lack motivation for tobacco cessation, that individuals with mental illness do not have the capacity to quit and that efforts to quit may actually impede their mental health treatment. Because of this misinformation, lack of awareness and smoking cessation interventions competence, tobacco use among this special group has been overlooked, depriving mental health patients of the necessary interventions for its control and prevention [17].

Few studies have focused on group behavioral interventions without addition of pharmacotherapies among this group, and few, if any, of these studies have been performed in Africa. In Kenya, there is limited access to tobacco cessation pharmacotherapies, which are also not affordable to most of this population. Because of the need, this study sought to evaluate the efficacy and safety of a group tobacco cessation behavioral intervention among patients with mental illness who attend outpatient programs at Mathari Referral and Teaching Hospital in Nairobi.

### Objectives

1. To determine the efficacy and safety of a group tobacco cessation behavioral intervention among tobacco using patients with mental illnesses on tobacco cessation at 6 months.
2. To evaluate the impact of tobacco cessation on health-related quality of life of patients with mental illnesses.

## Methods/ design

### Study design

This study used a controlled clinical trial design.

### Study setting

This study was conducted at Mathari Referral and Teaching Hospital Clinic for Substance Abuse Treatment (CSAT) and outpatient follow-up clinics. Mathari Referral and Teaching Hospital is Kenya’s only national referral and teaching psychiatric hospital with a capacity of 700 beds. The staff who provide services at the hospital include 243 nurses, 7 psychiatrists (2 of whom are full-time administrators), 2 pharmacists, and support staff. Tobacco cessation therapy was provided by trained tobacco cessation counsellors. The study was conducted at the CSAT and other outpatient clinics.

### Sample size calculation [18]

The sample size necessary for comparing proportions includes: proportion *p*_*1*_ being the estimated proportion of study participants with improved health status after the intervention and *p*_*2*_ being the estimated proportion of study participants with improved health outcomes in the control group.

$$ n=\left(\frac{r+1}{r}\right)\;\frac{\left(\overline{p}\right)\;\left(1-\overline{p}\right)\;{\left({Z}_{\beta }+{Z}_{\alpha /2}\right)}^2}{{\left({p}_1-{p}_2\right)}^2} $$

**Table:** 

Ratio of intervention: controls (r)	1
Estimated proportion with improved outcomes in intervention group p_1_	35.0%
Estimated proportion with improved outcomes in control group p_2_	15.0%
Odds Ratio	3.05
Average of proportions (p_1_ + p_2_)/2	0.25
Power (1-α/2)	95%
Power (1-β)	80%
Sample size per group	37
Sample size per group after adjusting for 30% attrition rate	48
**Total sample size**	**96**

### Inclusion criteria

1. 18 years of age and above.
2. History of tobacco use for more than 6 months.
3. A Fagerstrom score of 6 and above, which is a threshold consistent with dependence [17].
4. Currently on outpatient follow up treatment for a diagnosed mental health condition.
5. Willing to be part of the study for 6 months.

### Exclusion criteria

1. Patients on nicotine replacement therapy (NRT) or other pharmacotherapy for tobacco cessation.
2. Patients currently experiencing severe psychotic episodes judged by their treating health care provider.
3. Patients who would not be able to commit to the group sessions, defined as those who would not be able to attend group sessions for any reason, including transport-related reasons.

### Recruitment

Prior to recruitment, YO trained 2 counsellors or nurses who assisted with recruitment, screening, intake, and registration. Three addiction therapists who did not work at the hospital were trained by YO to lead tobacco cessation groups tailored to psychiatric patients. All patients who were enrolled for follow-up programs at CSAT and follow-up clinics were asked about their history of tobacco use by the intake/registration nurses.

All patients who walked in for follow-up after hospital discharge for a primary mental health diagnosis were informed of the study and were invited to participate. Informed consent was obtained from individuals who had capacity to provide informed consent in the presence of the clinicians working with the participants to ensure they understood the study requirements. Individuals who did not have capacity to provide informed consent were not recruited to minimize potential risks to this vulnerable population. After providing informed consent, participants were asked to complete the Fagerstrom tobacco use test. Participants were then asked to complete sociodemographic questionnaire and the World Health Organization (WHO) quality of life questionnaire. This screening and recruitment continued until the number of participants who provided informed consent reached 100.

### Randomization/allocation

Participants were recruited in clusters of 10 for allocation into the intervention and control groups. The first 10 participants formed group 1, and the next 10 participants formed group 2. Group 1 became the first intervention group, while group 2 became the first control group. This procedure continued until all 10 groups were formed (5 intervention and 5 control groups).

Participants were followed up clinically for ongoing mental health care on their regular clinic days.

### Study intervention

5As-based brief advice was offered to the intervention group participants by the study team who were trained by YO. This brief advice consisted of an individual session lasting approximately 5 min for each participant immediately after their consent had been obtained. The focus of the 5As was to enable the therapist know the immediate concern of each participant and to enable adequate support when the particular issues were raised during the group intervention sessions. The behavioral group intervention consisted of 6 sessions over 12 weeks and were led by 2 trained facilitators, followed by monthly group meetings from weeks 14 to 26. This program was adapted from the Royal Australian College of General Practitioners’ *Supporting Smoking Cessation Guide for Health Professionals* [19] and the World Health Organization’s *Strengthening Health Systems for Treating Tobacco Dependence in Primary Care* training package [20].

The topics that were explored during the group sessions include:

1. Introduction to the Program and Reasons to Quit
2. Benefits of Quitting and Understanding Why We Smoke and Ways of Quitting
3. Withdrawal Symptoms and Social Support
4. Dealing with Stress and Anxiety and Coping with Depression due to withdrawal
5. Assertiveness Training and Anger Management
6. Tobacco-Free Lifestyle and Dealing with High Risk Situations

#### Group session 1 (week 1)

On the first session, participants were introduced to the study program and specific components of the group behavioral intervention. Participants shared their expectations and experiences in their goal of tobacco cessation. The estimated time for group session 1 was 30–45 min.

#### Group sessions 2–6 (weeks 2–11)

Participants set their anticipated quit date on the 2nd week, which was their second session. During weeks 2 through 11, before the start of the session, feelings of participants were explored, the previous week’s self-reported tobacco consumption or cessation attempt was recorded. The topic of each week was explored first by lecture to explain the topic, and then group members took turns sharing their experiences on the topic.

#### Group sessions 7–9 (weeks 14–26)

Participants continued attending the CSAT outpatient and ward follow-up programs during this period. Behavioral group sessions 7–9 (weeks 14–26) were conducted once a month by the facilitators whereby each session was begun with a round of discussion on how participants were feeling about their cessation attempts, including any challenges they had experienced. The self-reported amount of tobacco used and quit attempts were documented. The study team also documented the challenges raised and tried to offer practical and supportive therapy for the challenges.

Summary of the group sessions and topics can be found in Table 1.

**Table 1:** Group sessions and topics summary

Time Frame	Group sessions
After signed consent	5 As Ask- History of tobacco use Advice- On need to quit tobacco use, and inform on effects of tobacco use Assess- On readiness to quit tobacco use, if not ready use motivational skills to increase readiness Assist- Offer intervention support. Inform on behavioral coping skills and available pharmacotherapies Arrange- Arrange date, time and place of next session to introduce coping skills)
Session 1 (Week 1)	7. Introduction to the program and reasons to quit tobacco use
Session 2 (Week 3)	8. Benefits of quitting and understanding why we smoke and ways of quitting 9. setting quit date
Session 3 (Week 5)	10. Withdrawal symptoms and social support
Session 4 (Week 7)	11. Dealing with stress and anxiety and coping with depression
Session 5 (Week 9)	12. Assertiveness training and anger management
Session 6 (Week 11)	13. Tobacco-free lifestyle and dealing with high risk situations
Follow up sessions (Weeks 14–26)	14. Round of discussion on participants’ feelings, cessation attempts, challenges experienced, and how they coped 15. Documentation of self-reported quit attempts 16. Supportive therapy

### Baseline and follow-up assessments

During baseline, Age of first use, duration of use, amount used, mental illness managed, Fagerstrom test score, and other substances used were documented. During weeks 4, 12 and 26, Quit attempts made, amount currently being smoked and Cotinine test results were documented. During week 26, the WHOQOL questionnaire was re-administered.

Based on previous tobacco cessation intervention trials, it was assumed that those who did not report abstinence had positive cotinine results.

At the group assessments (Weeks 4, 12, and 26), participants who reported continued tobacco use abstinence and consented to a saliva test were tested using a nicotine cotinine strip (Devon Medical: Nicotine/tobacco test kit). The saliva tests were evaluated by the nurses working at the hospital who were blinded to treatment allocation and were not otherwise part of the study.

The summary of the follow-up assessment activities is reported in Table 2.

**Table 2:** Baseline, Follow-up activities and Data to be collected summary

Follow-up time	Activity	Documentation
Week 0 (Baseline)	• Socio-demographic questionnaire • Fagerstrom test for nicotine dependence • WHO quality of life (QOL) instrument	• Age of first use, duration of use, amount used, mental illness managed, other substances used • Fagerstrom test score • WHO QOL score
Week 4	• Explored progress made and documented quit attempts • Focused on challenges experienced and how they were managed • Documented number of sticks currently smoked • Those reporting complete cessation and who gave consent for saliva testing, were then screened using cotinine test	• Quit attempts made • Amount currently being smoked • Cotinine test
Week 12	• Explored progress made and documented quit attempts • Focused on challenges experienced and how they were managed • Documented number of sticks currently smoked • Those reporting complete cessation and who gave consent, were screened using cotinine test	• Quit attempts made • Amount currently being smoked • Cotinine test
Week 26 (Final follow-up)	• Explored progress made and documented quit attempts • Focused on challenges experienced and how they were managed • Documented number of sticks currently smoked • Those reporting complete cessation, gave consent, then were screened using cotinine test • Administered WHO QOL instrument	• Recorded quit attempts made • Amount currently being smoked • Cotinine test • WHO QOL score

### Control group procedures

The control group was provided questionnaires to fill at the end of Weeks 4, 12, and 26. During the rest of the study, they continued receiving usual care, including clinical care at CSAT. After the conclusion of the study, the control group will be offered the group sessions that the intervention group received.

### Outcome measures

The study’s primary outcome was self-reported continuous tobacco use abstinence, biochemically verified, at Week 26. It was estimated that the intervention would increase tobacco cessation from the current rate of 15 to 35% [21]. While adjusting for 30% attrition rate, with a sample size of 48 per group, 96 participants would provide 80% power to detect this between-group difference with a two-sided alpha = 0.05.

Secondary outcomes assessed included cessation at Week 4 and 12, number of quit attempts, reduction in number of cigarettes used per day, and health-related quality of life measured at Week 26.

### Research instruments

#### 5As: stages in the process of smoking cessation

The tobacco cessation research literature strongly supports the use of a comprehensive, clinic-based approach to tobacco cessation, known as the 5A’s— ask, advise, assess, assist, and arrange follow-up. Despite rates of performance of the ask, assess, and advise components of the 5A’s model increasing and being effective, helping and arranging for follow-up components, are far less common [22].

#### Fagerstrom test

The Fagerstrom Test for Nicotine Dependence (FTND) is a widely used and researched short questionnaire [23]. The FTND has been found to have good test-retest reliability, convergent validity, and discriminant validity and performs well from a psychometric perspective with smokers with mental illness as it does with smokers without mental illness [24]. Several studies analysis on the reliability index for the overall score on the FTND indicated that it was excellent (0.87). In 14 studies that evaluated the internal consistency of the FTND, the Cronbach’s alpha coefficient ranged from 0.55 to 0.74, indicating that the FTND has moderate internal consistency [25]. The FTND can be administered in an interview or tobacco users can self-administer the questionnaire. The score ranges from 0 to 10 points, and the mean score of representative samples of smokers is usually in the range of 3–4 points [26].

#### World Health Organization quality of Life-100 questionnaire

The World Health Organization developed the World Health Organization Quality of Life-100 (WHOQOL-100) as a cross-cultural method of assessing QOL separate from a specific disease. Defining quality of life as an “individual’s perceptions of their position in life in the context of the culture and value systems in which they live, and in relation to their goals, expectations, standards, and concerns” [27], the WHOQOL-100 allows the respondent to determine his or her satisfaction while limiting constraints from cultural expectations or developer biases.

The WHOQOL-100 has been validated for use in a mental health population [28].

### Participants’ incentives

Participants were reimbursed Kshs. 200 (approximately USD $2) for transport and time spent at the hospital because of the study.

### Project team

- YO was the lead investigator and trained the research assistants, nurses, and counselors who were part of the study.

- 2 nurses who were part of the hospital staff, who were working at the outpatient registry were recruited to assist in identification of all patients who use tobacco and screened them for eligibility for the study.
- 2 counselors who were not part of the hospital team were trained by YO in tobacco cessation group therapy to lead in facilitation of the group sessions and document the progress and findings.

### Ethical considerations

The research proposal was presented to the department of Psychiatry University of Nairobi (UoN) for study clearance approval. The study and associated protocol were approved by the ethical committee of Kenyatta National Hospital in February 2017. The trial and its protocol were registered in May 2018 by the National Commission for Science, Technology and Innovation (NACOSTI); Permit No: NACOSTI/P/18/37962/21104.

Eligible participants for this study were those who were mentally stable and were in a position to provide informed consent. For those eligible participants who did not understand English nor Kiswahili, a translator was availed for them from a member of the research team trained in responsible conduct of research. If a translator was not readily available, then the participant was given another date to come back when a translator would be available. All participants were briefed on the purpose of the study. The study did not anticipate any more than minimal risk to the participants through their participation because those who had serious withdrawals, as described by the participants, would not be included and would be referred for appropriate interventions. YO is a trained mental health specialist, has been trained in research among vulnerable populations, oversaw the study interventions on site, and participated in facilitating the interventions.

Each screened individual was assured of confidentiality and allowed to ask questions or clarifications prior to participating in the study. Also, participants were enrolled using their client codes to protect their identities, and any identifying information was stored in a password-protected, securely stored file by YO. Considering that the study asked questions that might have made the respondents uncomfortable, those who chose to participate were informed of these risks during the informed consent process for the study, and they were also informed that they had the right to withdraw at any time without any repercussions. The study had safety precautions for participants such that any participant who was found to need further psychological services (e.g. severe withdrawal, confusion, or some form of psychosis) would be referred to the appropriate clinical program or facilities where necessary or advised to seek further management. No such measures were needed.

Dissemination of results is planned through publications in peer-reviewed journals and presentations in scientific conferences. Results will also be disseminated through presentations in local mental health meetings and within the study site. Data sharing maybe considered upon reasonable request.

### Data management and analysis

Data were collected using structured questionnaires and entered into a secure, password-protected database. The hard copy data forms have been stored in a lockable cabinet in lockable office during collection, data entry, analysis, and after study completion. Upon completion of data entry, 20% of hard copy forms will be compared with the entered data to identify systematic or random errors and corrections and repeated data entry will be made appropriately, including 100% recheck if a > 2% error rate is detected.

Sociodemographic characteristics of the study participants, such as age, gender, residence, education level, occupation and perceived health status will be reported. We will also report participants’ health-related quality of life at baseline using the WHOQOL-100 and document participants’ primary mental health disorder. The kind of tobacco product under use, daily amount of tobacco consumption, duration of use, and age of initiation to tobacco will also be described. Using the Fagerstom test, we will determine nicotine dependency and describe intent to quit use of tobacco products. Data on any other substance use such as alcohol, bhang and miraa, including duration of use, will also be reported.

During exploratory data analysis, discrete variables will be summarized using frequencies and percentages, while continuous variables will be summarized using measures of central tendency and dispersion such as means with standard deviations and median with inter-quartile ranges, where appropriate. Bivariate analysis will be carried out to compare the intervention groups with the control groups with respect to socio-demographic characteristics, history of tobacco and substance abuse, type(s) of mental illness, and WHOQOL-100 scores at baseline. Mann Whitney tests will be employed to compare the intervention and control groups where predictor variables are continuous, and chi-squared tests will be applied when the predictor variable is categorical. We will compare unadjusted and adjusted cessation rates at 6 months, recognizing the limitations of the study design at eliminating potential confounders between the two groups given the non-randomized nature of group allocation. Potential confounders include age, sex, baseline tobacco, and baseline type of mental illness, among others. Time to cessation of tobacco use will be compared between the two groups by creating Kaplan-Meier curves and with the creation of corresponding Cox proportion hazard models to determine the effect of the intervention on the primary study outcome. We will report unadjusted and adjusted models, the latter which will aim to control for independent factors associated with relapse. We will perform similar analyses for secondary outcomes as well. All analyses will be carried out using IBM SPSS Statistics Software Version 24, and a two-sided *p* < 0.05 will be used to determine statistical significance.

## Discussion

This group tobacco cessation behavioral intervention among persons with mental illness seeks to identify a safe and effective intervention for this high-burden outpatient population in Kenya. The study methodology integrated the normal clinic attendance days and time to ensure convenience for the participants as well as no extra expense incurred. The goal is to demonstrate the feasibility of this accessible intervention to patients in need of accessible and affordable treatment options. This study intervention may guide cessation interventions among this group, as well as national and regional discussions among the low- and middle-income countries with limited access to tobacco cessation pharmacotherapies. It may also highlight the potential value of integrated cessation services, which might be safe, effective, cost-effective, and affordable, as well as the potential value of training of healthcare providers in tobacco cessation intervention among this population.

### Strengths of the study

1. This is the first study focusing of its kind on this population that has been done in Kenya
2. Study was carried during normal clinic days, which encourages participation
3. Use of group sessions that allowed participants to share their experiences (both good and bad) and normalize them.
4. Followed up participants for 3 months after the intervention.
5. The use of the hospital’s clinicians during recruitment which made for familiar faces and made encouraged trust between the participants and the researchers.

### Limitations of the study

1. The study did not explicitly ask about the participants desire to quit prior to recruitment.
2. The study did not offer medication to participants with severe nicotine withdrawal; it referred them appropriately but they were discontinued from the study.
3. Not all patients who wanted to quit tobacco use were recruited; only those who scored 6 on the Fagerstrom Nicotine test were recruited.
4. Under 18 years with nicotine dependence and a desire to quit were not enrolled in the study

### Current status

The trial started recruiting participants on 10th April 2017, with follow-up completed on 28th June 2019. Trial results will be available in August 2019.

## Data Availability

Data sharing may be considered upon reasonable request.

## Abbreviations

CSAT: Clinic for Substance Abuse Treatment
DSM: Diagnostic and Statistical Manual of Mental Disorders
FTND: Fagerstrom Test for Nicotine Dependence
ICD: International Classification of Diseases
NACOSTI: National Commission for Science, Technology and Innovation
NRT: Nicotine Replacement Therapies
UoN: University of Nairobi
WHO: World Health Organization
WHOQOL: World Health Organization Quality of Life

## References

[1] World Health Organization. International statistical classification of diseases and related health problems, 10th revision, Fifth edition, 2016. World Health Organization. 2015. https://apps.who.int/iris/handle/10665/246208.

[2] American Psychiatric Association (2013). Diagnostic and Statistical Manual of Mental Disorders (Fifth version).

[3] Action on Smoking and Health (ASH) Factsheet. Smoking and Mental Health. 2016. Available at: http://ash.org.uk/category/information-and-resources/fact-sheets/. Accessed 15 May 2019.

[4] Heiligenstein E, Smith SS (2006). Smoking and mental health problems in treatment seeking university students. Nicotine Tob Res.

[5] Brown C (2004). Tobacco and mental health: a review of the literature.

[6] Royal College of Psychiatrists. Liaison Psychiatry for acute hospital: Integrated mental and physical healthcare. London: College Report, Royal College of Psychiatrists. 2013.

[7] John U, Meyer C, Rumpf HJ, Hapke U (2004). Smoking, nicotine dependence and psychiatric comorbidity: a population-based study including smoking cessation after three years. Drug Alcohol Depend.

[8] Weir K (2013). Smoking and mental illness. American Psychological Association Science Watch.

[9] World Health Organization. Division of Mental Health. WHOQOL-BREF: introduction, administration, scoring and generic version of the assessment: field trial version, December 1996: World Health Organization; 1996. https://apps.who.int/iris/handle/10665/63529

[10] Katsching H, Freeman H, Sarorius N (1997). Quality of life in mental disorders. Am J Psychiatr.

[11] Hall S, Prochaska JJ (2009). Treatment of smokers with co-occurring disorders: emphasis on integration in mental health and addiction treatment settings. Annu Rev Clin Psycho.

[12] Cavasoz-Rehg PA, Breslau N, Hatsukami D, Krauss MJ, Spitznagel EL, Grucza RA, Salyer P, Hartz SM, Bierut LJ (2014). Smoking cessation is associated with lower rates of mood/anxiety and alcohol use disorders. Psychol Med.

[13] Prochaska JJ, Delucchi K, Hall SM (2004). A meta-analysis of smoking cessation interventions with individuals in substance abuse treatment or recovery. J Consult Clin Psychol.

[14] Gulliver SB, Wolfsdorf BA, Morissette SB (2004). Treating tobacco dependence: development of a smoking cessation treatment program for outpatient mental health clinics. Cogn Behav Pract.

[15] Morris CD, Tedeschi GJ, Waxmonsky J, May M, Giese A (2009). Tobacco quit lines and persons with mental illnesses: perspective, practice and directions. J Am Psychiatr Nurses Assoc.

[16] Tsoi DT, Porwal M, Webster AC. Interventions for smoking cessation and reduction in individuals with schizophrenia. Cochrane Database Syst Rev*, Issue* 2. 28,Feb 2013; (2):CD007253. 10.1002/14651858.CD007253.pub3 PMC6486303 23450574

[17] Bron C, Zullino D, Besson J, Borgeat F (2000). Smoking in psychiatry, a neglected problem. Praxis (Bern 1994).

[18] Reference: Cochran WG (1963). Sampling Techniques. 2nd ed. New York: John Wiley and Sons, Inc.,

[19] The Royal Australian College of General Practitioners. Supporting smoking cessation: Guide for health professionals. Royal College of General Practitioners. Melbourne, Australia. 2011.

[20] World Health Organization. Strengthening health systems for treating tobacco dependence in primary care: Building capacity for tobacco control training package. World Health Organization. 2013. https://apps.who.int/iris/handle/10665/84388.

[21] Cochran WG (1963). Sampling techniques 2nd ed.

[22] Bentz CJ, Bayley KB, Bonin EK (2006). Provider feedback to improve 5As tobacco cessation in primary care: a cluster randomized clinical trial. Nicotine and Tobacco Research.

[23] Fagerstrom Karl -Oiov, Schneider Nina G. (1989). Measuring nicotine dependence: A review of the Fagerstrom Tolerance Questionnaire. Journal of Behavioral Medicine.

[24] Buckley T, Mozley M (2004). SL et al. a psychometric evaluation of the Fagerstrom test for nicotine dependence in PTSD smokers. Addict Behav.

[25] Meneses-Gaya IC, Zuardi AW, Loureiro SR, Crippa JAC (2009). Systematic review: psychometric properties of the Fagerstrom test for nicotine dependence. Bras Pneumol.

[26] World Health Organization. Study protocol for the World Health Organization project to develop a quality of life assessment instrument (WHOQOL). Qual Life Res. 1993:153–9. 8518769

[27] Mas-Expósito Laia, Amador-Campos Juan Antonio, Gómez-Benito Juana, Lalucat-Jo Lluís (2011). The World Health Organization Quality of Life Scale Brief Version: a validation study in patients with schizophrenia. Quality of Life Research.

[28] Fagerstrom KO, Schneider NG (1989). Measuring nicotine dependence: a review of the Fagerstrom tolerance questionnaire. J Behav Med.

